# Comparing the Recombinant Protein Production Potential of Planktonic and Biofilm Cells

**DOI:** 10.3390/microorganisms6020048

**Published:** 2018-05-24

**Authors:** Alexandra Soares, Luciana Calheiros Gomes, Filipe José Mergulhão

**Affiliations:** LEPABE—Department of Chemical Engineering, Faculty of Engineering, University of Porto, Rua Dr. Roberto Frias, 4200-465 Porto, Portugal; asoares@fe.up.pt (A.S.); luciana.gomes@fe.up.pt (L.C.G.)

**Keywords:** green fluorescent protein, *Escherichia coli*, biofilm, planktonic, flow cell reactor

## Abstract

Recombinant protein production in bacterial cells is commonly performed using planktonic cultures. However, the natural state for many bacteria is living in communities attached to surfaces forming biofilms. In this work, a flow cell system was used to compare the production of a model recombinant protein (enhanced green fluorescent protein, eGFP) between planktonic and biofilm cells. The fluorometric analysis revealed that when the system was in steady state, the average specific eGFP production from *Escherichia coli* biofilm cells was 10-fold higher than in planktonic cells. Additionally, epifluorescence microscopy was used to determine the percentage of eGFP-expressing cells in both planktonic and biofilm populations. In steady state, the percentage of planktonic-expressing cells oscillated around 5%, whereas for biofilms eGFP-expressing cells represented on average 21% of the total cell population. Therefore, the combination of fluorometric and microscopy data allowed us to conclude that *E. coli* biofilm cells can have a higher recombinant protein production capacity when compared to their planktonic counterparts.

## 1. Introduction

Recombinant proteins are currently used in different biotechnological industries and are produced in large amounts in bioreactors [1]. An important step in recombinant protein production is to choose the ideal expression system and factors like protein quality, functionality, production speed, and yield should be considered [2]. *Escherichia coli* has been widely used for the production of recombinant proteins due to its fast growth at high cell densities, minimal nutrient requirements, well-characterized genetics, and the availability of a large number of cloning vectors [2,3]. In the early 1980s, the Food and Drug Administration (FDA) approved the first recombinant insulin produced in *E. coli* [4] and since then this bacterium is one of the organisms of choice for the production of several commercial recombinant proteins [5].

Most of the research on the expression of recombinant proteins has been based on planktonic bacteria grown in liquid cultures. However, the natural state for many bacteria is to live in communities of sessile cells forming biofilms [6]. Biofilms are communities of surface-attached microorganisms encased in a self-produced extracellular matrix [7]. Such biological organization provides distinct characteristics to bacteria compared to their planktonic counterparts [8]. For instance, local environmental conditions arising as a result of mass transport limitations, intercellular signaling, and other phenomena, may induce biofilm cells to modulate expression of genes differently than in suspended populations [9]. The expression of recombinant proteins in *E. coli* is commonly accomplished by inserting the gene of interest into a multicopy plasmid [10] that imposes a metabolic burden on the host cell [11]. In planktonic cells, this added metabolic burden may decrease cellular growth rates and biomass yields [11], particularly when the plasmid vector is used for the direct production of a recombinant protein [12]. Conjugative [13,14,15,16,17] and non-conjugative plasmids [18,19,20,21] were shown to increase biofilm formation. For example, our research group has previously shown that the presence of the non-conjugative plasmid pET28A in *E. coli* JM109(DE3) cells increased biofilm formation when compared to a non-transformed strain [18]. 

In 2007, O’Connell et al. [22] examined the production of eGFP in a chemostat with planktonic cells and in a parallel plate flow cell (PPFC) reactor with biofilm cells. This was the first experiment showing a high-level production of a heterologous protein in *E. coli* biofilms. Although the number of studies on the effect of recombinant protein expression on sessile cells is still scarce, it has been suggested that the biofilm environment benefits recombinant protein production [18,23]. 

The aim of this work was to compare the production of a model recombinant protein (enhanced green fluorescent protein, eGFP) between *E. coli* planktonic and biofilm cells. In the present study, the comparison between planktonic and biofilm cells was explored using a novel single-cell methodology which combines the common epifluorescence microscopy with an image analysis tool. Additionally, the plasmid maintenance in both planktonic and sessile cells was also assessed for the first time.

## 2. Materials and Methods 

### 2.1. Bacterial Strain

The *E. coli* strain JM109(DE3) from Promega (Madison, WI, USA) was transformed with the plasmid pFM23 (constructed from pET28A vector; Novagen, Madison, WI, USA) for the cytoplasmic production of eGFP [24]. This plasmid contains a kanamycin resistance gene and a pMB1 origin of replication (medium-copy number replicon), and uses the T7 promoter for transcription of the foreign gene, which can be induced by lactose or its non-hydrolyzable analogue isopropyl β-d-1-thiogalactopyranoside (IPTG).

### 2.2. Flow Cell System and Experimental Conditions

A reactor system comprising a recirculating tank (for planktonic cells), a vertical flow cell reactor (where biofilms are formed), and peristaltic and centrifuge pumps that allow the circulation of the bacterial suspension was operated as described by Gomes et al. [25]. The flow cell reactor is a semi-circular Perspex duct with apertures on its flat wall to fit removable Perspex pieces (coupons) to which polyvinyl chloride (PVC) slides were glued. The biofilms were developed on the upper faces of the PVC slides that were in contact with the bacterial suspension circulating through the system. 

*E. coli* cells were grown by recirculating the bacterial suspension at 30 °C for 11 days under turbulent flow (Reynolds number of 4600) [25]. The recirculating tank was aerated using an air pump (flow rate of 108 L h^−1^) and continuously fed with lysogeny medium (LB-Miller; Sigma, St. Louis, USA) supplemented with 20 μg mL^–1^ kanamycin at a flow rate of 0.025 L h^–1^ [25].

### 2.3. Biofilm and Planktonic Monitoring

The system was stopped daily and a coupon was carefully removed from the flow cell for biofilm sampling. The biofilm wet weight and thickness were firstly determined by comparing the coupon weight prior to the start of the experiment and on the sampling day, and by using a digital micrometer (VS-30H; Mitsubishi Kasei Corporation, Tokyo, Japan) [26], respectively. Then, the biofilm was scrapped off the coupon using a pipette tip and resuspended in 25 mL of sterile saline solution (0.85% NaCl) for total and viable cell quantification and eGFP analysis. 

Biofilm total (viable plus non-viable) and viable cell counts were determined using the Live/Dead^®^ BacLight^TM^ bacterial viability kit (Syto9/propidium iodide; Invitrogen Life Technologies, Alfagene, Carcavelos, Portugal) as fully described in Gomes et al. [25]. Briefly, bacterial cells from the biofilm suspension were filtered through a Nucleopore Track-Etch Membrane of black polycarbonate (pore size of 0.22 µm; Whatman Ltd., Buckinghamshire, UK), stained for 10 min in the dark and observed using a Leica DM LB2 epifluorescence microscope connected to a Leica DFC300 FX camera (Leica Microsystems Ltd, Heerbrugg, Switzerland). The image processing software ImageJ v1.48 (National Institutes of Health, Bethesda, MD, USA) was used to estimate the number of viable and non-viable cells on each membrane from counts of a minimum of 15 fields of view. The final values of biofilm total cells were expressed as log cells cm^−2^ of coupon area and the percentage of biofilm viability on each day was calculated by dividing the viable cell counts by the total cell number. 

The eGFP expression in biofilm cells was assessed by both epifluorescence microscopy [25] and fluorometry [24]. For the microscopic method, the biofilm suspension was filtered and observed using a Leica DM LB2 epifluorescence microscope (Leica Microsystems Ltd., Heerbrugg, Switzerland). Fifteen fields of view were photographed for each sample in order to estimate the number of eGFP-expressing cells [25]. The percentage of eGFP-expressing cells in the biofilm was then calculated by dividing the number of eGFP-expressing cells by the total number of cells. The specific eGFP production in biofilms was quantified by the fluorometric method fully described by Mergulhão and Monteiro [24]. Briefly, a volume of biofilm detached cells corresponding to an equivalent optical density (OD) of 1 at 610 nm was centrifuged. The pellet was resuspended in 100 µL of Buffer I (50 mM Na_2_HPO_4_, 300 mM NaCl, pH 8) and added to 100 µL of Buffer I in a 96-well microtiter plate (Orange Scientific, Braine-l’Alleud, Belgium). Fluorescence was measured in a microplate reader (SpectraMax M2E; Molecular Devices, Inc., Berkshire, UK) with a 488 nm-excitation filter and a 507 nm-emission filter. Calibration curves were constructed with purified eGFP and the final values were presented as specific eGFP production (fg cell^−1^). An OD_610nm_ of 1 corresponds to a cellular concentration of 7.6 × 10^8^ cells mL^−1^.

**Figure 1:**
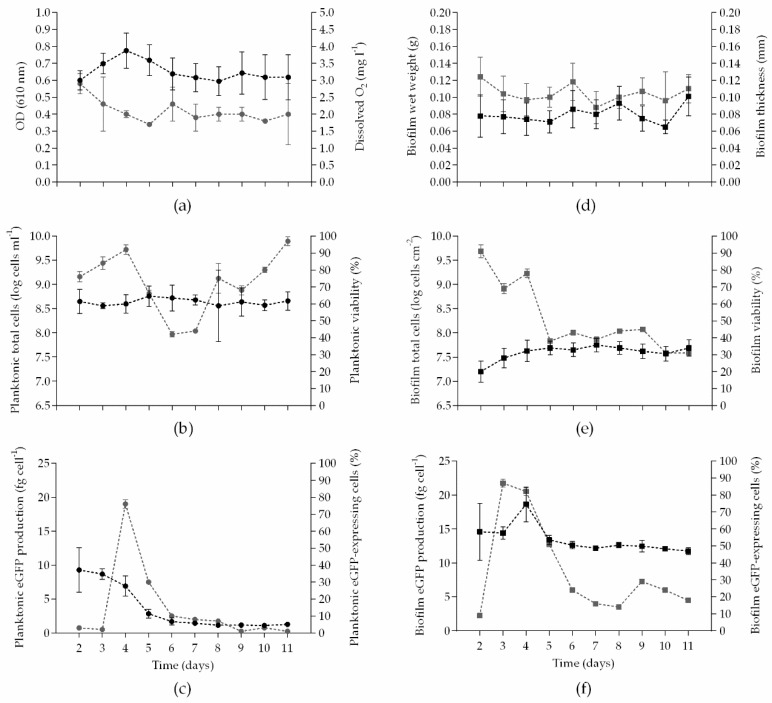
Time-course evolution of planktonic and biofilm parameters: (**a**) OD_610nm_ and dissolved oxygen in the recirculating tank; (**b**) planktonic total cells and planktonic viability; (**c**) planktonic eGFP production and percentage of planktonic eGFP-expressing cells; (**d**) biofilm wet weight and thickness; (**e**) biofilm total cells and biofilm viability; (**f**) biofilm eGFP production and percentage of biofilm eGFP-expressing cells. Black circles (●) and squares (■) on the left y-axis, and grey circles (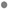) and squares (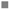) on the right y-axis. The means ± SDs for three independent experiments are illustrated.

For monitoring the planktonic cells, a sample was taken from the recirculating tank to first assess the OD_610nm_ and the dissolved oxygen (OX 4100 H; VWR, Alfragide, Portugal). Total and viable cell counts, eGFP-expressing cell counts and specific eGFP production were also assessed as previously described for the biofilm cells (Figure 1). 

### 2.4. Measurement of Plasmid Maintenance

Planktonic and biofilm sample suspensions were suitably diluted with saline solution and spread on both selective (supplemented with 20 μg mL^–1^ kanamycin) and non-selective agar plates (Plate Count Agar, Merck, Lisboa, Portugal) to form between 10 and 300 colony forming units (CFU) per plate. Colony enumeration was carried out after 24-h incubation at 30 °C. The number of CFU on selective plates corresponds to the number of culturable cells carrying the plasmid pFM23, whereas the CFU detected on non-selective plates are equivalent to the total number of culturable cells, i.e., plasmid-bearing cells plus plasmid-free cells (see Supplementary Material, Figure S1). The maintenance of plasmid pFM23 in both planktonic and biofilm cells was determined as the ratio between the number of plasmid-bearing cells and the total number of culturable cells.

### 2.5. Statistical Analysis 

The results presented in Figure 1 originated from averages of triplicate sets obtained in independent experiments. Standard deviations (SDs) on the triplicate sets were calculated for each day and parameter. For planktonic growth (Figure 1a,b), the following SD averages were obtained: SD < 16% for OD_610nm_ and dissolved oxygen, SD < 4% for total cell counts, and SD < 5% for planktonic viability. Concerning biofilm formation (Figure 1d,e), the following SD averages were obtained: SD < 23% for biofilm wet weight, SD < 20% for biofilm thickness, SD < 3% for biofilm total cells, and SD < 4% for biofilm viability. Regarding the eGFP quantification (Figure 1c,f), SD < 19% for planktonic eGFP production, SD < 4% for planktonic eGFP-expressing cells, SD < 8% for biofilm eGFP production, and SD < 4% for biofilm eGFP-expressing cells were obtained.

Paired *t*-test analysis was performed based on a confidence level of 95% (differences reported as significant for *p* values < 0.05).

## 3. Results

In this work, two fluorescence-based techniques (fluorometry and epifluorescence microscopy) were used with the aim of comparing the eGFP production in planktonic and biofilm populations grown in a flow cell system.

Planktonic growth was monitored over 11 days by measuring the OD_610nm_ and dissolved oxygen in the recirculating tank (Figure 1a) and by quantifying the total cell number and cell viability (Figure 1b). The OD_610nm_ increased until day 4, which is in agreement with the corresponding decrease in the oxygen concentration in the recirculating culture over the same experimental period. Between days 4 and 6, the OD_610nm_ decreased and then stabilized from day 6 onwards. The same stabilization was observed in the oxygen consumption profile with dissolved oxygen levels around 2 mg L^−1^, thus it can be concluded that the flow cell system was in steady state between days 6 and 11. Looking at Figure 1b, the total number of planktonic cells remained practically constant over the experiment. However, the percentage of viability varied significantly (Figure 1b) due to oscillations on the number of viable cells during the experimental time (data not shown). Between days 4 and 6, a strong decrease in planktonic viability (from 92% to 42%) was observed. But from day 8 onwards, the planktonic viability increased significantly, reaching almost 100% at the end of the experiment. 

Regarding the biofilm growth, it was monitored by determining the wet weight and thickness (Figure 1d), and by quantifying the total cell number and cell viability (Figure 1e), as performed for planktonic cells. Biofilm wet weight remained practically constant during the experimental time, but a slight increase was observed in the last day of the experiment. The evolution of biofilm thickness was similar to the wet weight with average values around 0.104 mm. Figure 1e shows that the total number of biofilm cells increased slightly between days 2 and 5, remaining constant until the end of the experiment. Concerning biofilm viability (Figure 1e), it is possible to observe a marked reduction in cell viability until day 5 (38%) that resulted in viability percentages of on average 39% until day 11. Note that lower viability percentages were found for biofilms (Figure 1e) when compared to planktonic population (Figure 1b). 

By analysing the specific eGFP production (Figure 1c,f), it can be seen that sessile cells produced more recombinant protein than those grown in planktonic state. The biofilm environment enhanced the specific eGFP expression about 10-fold when compared to planktonic cells in steady state (statistically significant differences were confirmed for all experimental days, *p* < 0.05). Additionally, epifluorescence microscopy was used to determine the percentage of eGFP-expressing cells in both planktonic (Figure 1c) and biofilm populations (Figure 1f). The percentage of eGFP-expressing cells in planktonic state started to increase at day 3, reaching a maximum value of 76% at day 4 and then decreasing abruptly to values around 5% (Figure 1c). The equivalent biofilm curve (Figure 1f) also shows an initial increase in the percentage of expressing cells (with a maximum value of 87% between days 3 and 4), followed by a strong reduction (of approximately 58%) until day 6. In steady state, the percentage of planktonic expressing cells oscillated around 5%, whereas for biofilms, eGFP-expressing cells represented on average 21% of total cell population. Combining this information with the fluorimetric data, it is possible to conclude that *E. coli* biofilm cells can have a higher recombinant production capacity when compared to their planktonic counterparts under the tested conditions. This may be related with the highest potential of *E. coli* biofilms in maintaining the plasmid pFM23 within cells. In fact, in planktonic cells, for which the total cell number was constant over the course of the experiment (Figure 1b), the frequency of plasmid-containing bacteria was on average 0.33, while in the biofilm this parameter rose to approximately 0.90 in steady state (from day 5 onwards, Figure S1). 

## 4. Discussion

In this study, the production of a model recombinant protein (eGFP) in *E. coli* planktonic and biofilm cells was evaluated by fluorometry (a bulk method) and epifluorescence microscopy (a single-cell method). 

We found that the specific eGFP production was lower in planktonic cells and this may have contributed to the higher viability of these cells when compared to biofilm cells. It is well documented that cells bearing a plasmid may suffer from a metabolic burden as cellular resources are being used for its replication and for the expression of plasmid-encoded genes [11,27,28]. In particular, a decreased cellular viability of plasmid-bearing cells and an increased protease activity are some stress signals suffered by these cells [29,30]. 

The expression of recombinant proteins in *E. coli* biofilms was first reported by Huang et al. [20,21,31], who studied the production of β-galactosidase in a chemostat and in a parallel plate flow. These authors found that the recombinant protein was successfully produced in biofilm cells, although at a lower level than in planktonic cells [20,31]. In contrast, our results show that the *E. coli* biofilm environment enhanced eGFP production when compared to planktonic cells. Furthermore, biofilms presented a higher frequency of plasmid-bearing cells than the planktonic population. These results corroborate those obtained in a previous study of O’Connell et al. [22] who produced eGFP in a chemostat (planktonic cells) and in a PPFC reactor (biofilms). The results of their work indicated that the biofilm environment enhanced both plasmid maintenance and cellular GFP concentrations when compared to planktonic cells. Continuous biofilm cultures for recombinant protein production can be more beneficial for retention of plasmid-bearing cells than chemostats [22] since cells in biofilms tend to grow more slowly than their planktonic counterparts [32], leading to fewer divisions and correspondingly less plasmid segregation. Other authors have also shown that the plasmid loss is more significant in planktonic populations [33,34,35]. More recently, our research group found that the volumetric productivity of the biofilms developed in the flow cell system is already within the range that can be obtained by conventional high cell density cultures, even before optimization of cultivation conditions [23]. 

During recombinant protein production, it is essential to monitor the amount of target protein produced in order to find the best processing conditions. The use of a fluorescent protein as a model recombinant protein enables bulk and single-cell quantification. Bulk production can be assessed by fluorometry, which is a highly sensitive, specific, and simple method with low instrumental costs when compared to other techniques, like flow cytometry, that is also used to follow recombinant protein expression [36,37]. On the other hand, single-cell analysis by epifluorescence microscopy can provide information about the heterogeneity level of protein expression within a cell population [38]. In this work, the combination of these two techniques was found to be very useful in monitoring recombinant protein production in both planktonic and sessile cells.

## 5. Conclusions

This study revealed that the specific recombinant protein production and the percentage of eGFP-expressing cells were higher in *E. coli* biofilms than in cell suspension, which was associated with the highest potential of biofilms in retaining the plasmid.

When combined with a bulk method such as fluorimetry, epifluorescence microscopy and the corresponding image analysis is an innovative tool for determining important production parameters in both planktonic and sessile cells, namely the number of cells expressing the recombinant protein within a bacterial population. The methodology presented here is a simple, fast and low-cost way of obtaining information about the heterogeneity level of protein expression within a cell population and it can be further used to find the best processing conditions for recombinant protein production in biofilm reactors. 

## Supplementary Materials

The following are available online at http://www.mdpi.com/2076-2607/6/2/48/s1, Figure S1: Planktonic (a) and biofilm (b) population dynamics of plasmid-bearing cells (●) and total culturable cells (plasmid-bearing cells plus plasmid-free cells) (○). The means ± SDs for three independent experiments are illustrated.
