# Supplementary material for: Comparing the Recombinant Protein Production Potential of Planktonic and Biofilm Cells
Source: Microorganisms. 2018 May 24;6(2):48. doi: 10.3390/microorganisms6020048 (PMC6027475; doi:10.3390/microorganisms6020048)
Supplement: Supplementary file 1 [file microorganisms-06-00048-s001.pdf]

Supplementary Material

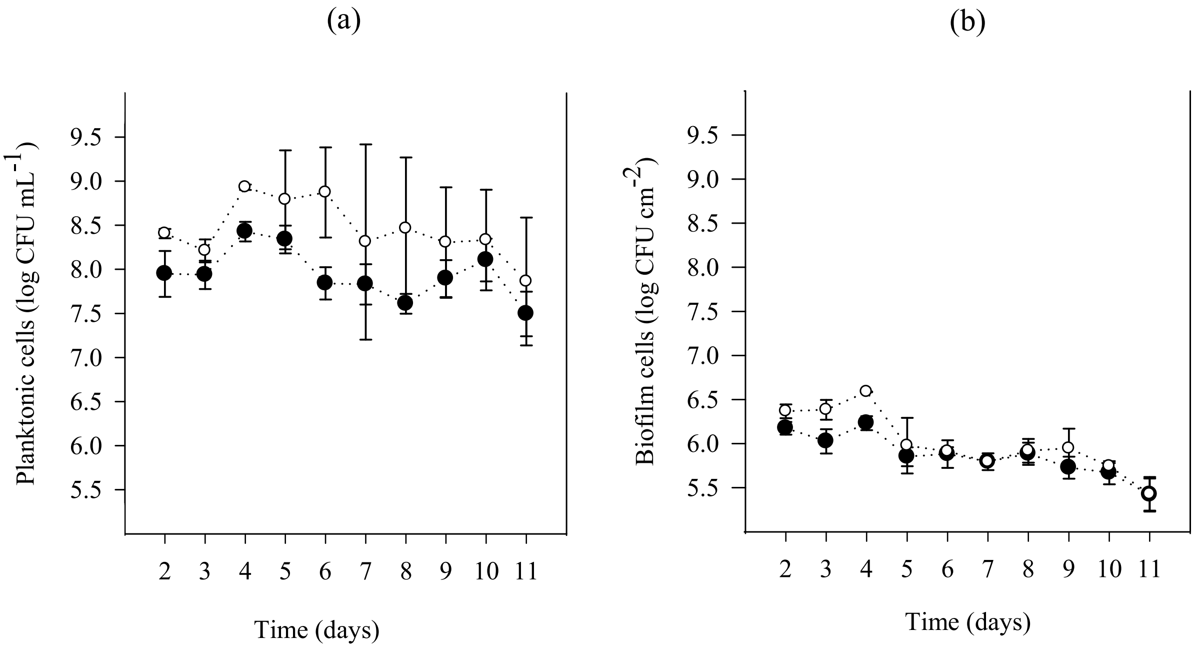

Figure S1. Planktonic (a) and biofilm (b) population dynamics of plasmid-bearing cells (●) and total culturable cells (plasmid-bearing cells plus plasmid-free cells) (○). The means ± SDs for three independent experiments are illustrated.
